# Short course of voriconazole therapy as a risk factor for relapse of invasive pulmonary aspergillosis

**DOI:** 10.1038/s41598-020-73098-w

**Published:** 2020-09-30

**Authors:** Dong Hoon Shin, Seung-Jin Yoo, Kang Il Jun, Hyungjin Kim, Chang Kyung Kang, Kyoung-Ho Song, Pyoeng Gyun Choe, Wan Beom Park, Ji-Hwan Bang, Eu Suk Kim, Sang Won Park, Hong Bin Kim, Nam-Joong Kim, Myoung-don Oh

**Affiliations:** 1 Department of Internal Medicine, Seoul National University College of Medicine, Seoul, Republic of Korea; 2 Department of Radiology, Seoul National University Hospital, Seoul, Republic of Korea; 3 Department of Internal Medicine, Seoul National University Bundang Hospital, Seongnam, Republic of Korea; 4 Department of Internal Medicine, Seoul Metropolitan Boramae Hospital, Seoul, Republic of Korea

**Keywords:** Fungi, Risk factors

## Abstract

To investigate associations of the duration of voriconazole treatment and radiological response with relapse of invasive pulmonary aspergillosis (IPA) in immunocompromised patients, we explored the risk factors for IPA relapse after successful initial treatment. All patients with proven or probable IPA who had finished voriconazole treatment between 2005 and 2019 in a tertiary-care hospital were reviewed. IPA relapse was defined as re-diagnosis of proven or probable IPA at the same site within 1 year after treatment termination. Short course of voriconazole treatment was defined as a treatment less than 9 weeks, which is a median of the recommended minimum duration of therapy from the Infectious Disease Society of America. The radiological response was defined as a reduction in IPA burden by more than 50% on chest computed tomography. Of 87 patients who had completed voriconazole treatment, 14 (16.1%) experienced IPA relapse. Multivariable Cox regression identified that short voriconazole treatment duration (adjusted hazard ratio [aHR], 3.7; 95% confidence interval [CI], 1.1–12.3; *P* = 0.033) and radiological non-response (aHR, 4.6; 95% CI, 1.2–17.5; *P* = 0.026) were independently associated with relapse of IPA after adjusting for several clinical risk factors. Longer duration of therapy should be considered for those at higher risk of relapse.

## Introduction

Invasive pulmonary aspergillosis (IPA) is an infectious disease with a mortality rate of about 30%^1,2^. Immunocompromised patients with prolonged neutropenia or taking corticosteroids, and those who received allogeneic hematopoietic stem cell transplantation (HCT) or solid-organ transplantation, and with severe influenza are at high risk of IPA^3,4^. Restoring immune function is crucial for treatment of IPA^3,5^.

Voriconazole is the drug of choice for IPA^1^. The recommended treatment duration is a minimum of 6–12 weeks, although there is little evidence for the optimal treatment duration^3^. In clinical practice, treatment is usually terminated by a physician’s subjective judgement on the patient’s immune status and treatment response. Therapeutic monitoring of IPA includes serial clinical evaluation of symptoms and signs, serial measurement of galactomannan assay (GM) titres and computed tomography (CT) scans^3^.

A fixed treatment duration for all patients is not possible, since treatment duration needs to be personalized and determined on a patient-basis. Nevertheless, a concern would be the minimum required duration of the voriconazole therapy^3,6,7^. Insufficient treatment may result in relapse of IPA, which would lead to the increased morbidity and mortality.

In this study, we hypothesized that short course of voriconazole treatment, defined empirically as less than 9 weeks (i.e., median of the commonly recommended duration by the Infectious Disease Society of America^3^), is associated with relapse of IPA. Therefore, in this retrospective cohort study from a single tertiary medical centre, we analyzed the association between the short treatment duration and relapse of IPA in immunocompromised patients after adjusting for the important clinical confounders using a regression analysis. In addition, we also investigated whether the radiological response at the time of treatment termination was associated with relapse of the disease.

## Results

### Clinical characteristics of patients with IPA

**Figure 1:**
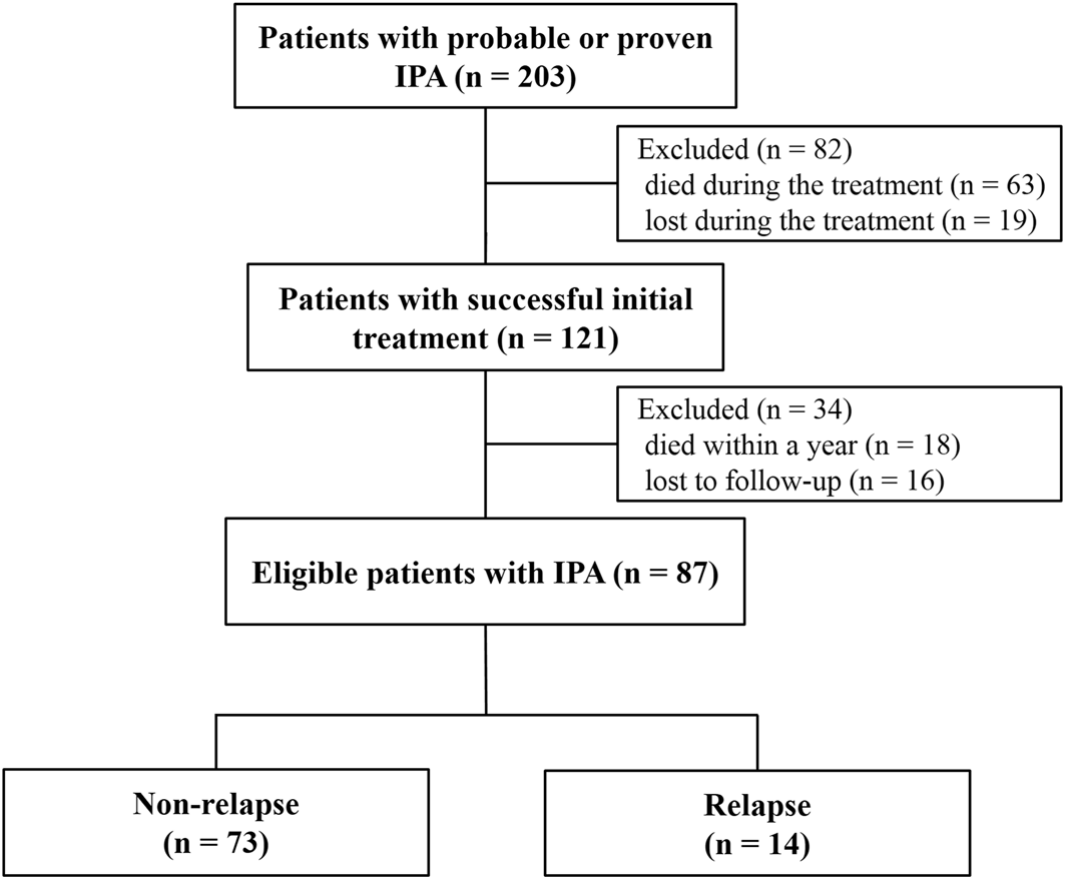
Flow diagram of the study. *IPA* invasive pulmonary aspergillosis.

Of 203 patients with proven or probable IPA, 121 had completed voriconazole treatment (Fig. 1). Among these, 34 died (n = 18) or were lost to follow-up (n = 16) within 1 year after treatment. Among all eligible patients (n = 87), 14 (16.1%) experienced IPA relapse. Median duration (interquartile range [IQR]) of voriconazole treatment was 18.9 (12.0–22.4) weeks. Voriconazole accounted for about 90% of the total anti-mold treatment period (median [IQR], 89.5 [86.1–100] in relapse group *vs.* 89.9 [84.2–100] in non-relapse group, *P* = 0.516). Median duration (IQR) from the end of treatment to the diagnosis of relapse was 10.8 (4.1–15.3) weeks.

### Comparison between patients with and without relapse

The clinical characteristics of the patients according to the relapse of IPA are described in Table 1. Diabetes mellitus was more common in the relapse group than in the non-relapse group (relapse vs. non-relapse; mean, 64.3% vs. 34.2%; *P* = 0.035), as was a higher Charlson’s comorbidity-weighted index score (mean [± standard deviation], 6.4 [± 1.4] vs. 4.8 [± 2.3]; *P* = 0.017). Duration of voriconazole treatment tended to be shorter in the relapse group (median week [IQR], 15.8 [8.2–21.5] vs. 19.5 [12.0–22.9]; *P* = 0.251). Two patients (5.7 weeks in relapse group and 4.7 weeks in non-relapse group, respectively) were treated for less than 6 weeks. Four (28.6%) and 7 (9.6%) patients underwent short course of voriconazole treatment in relapse and non-relapse groups, respectively (*P* = 0.063).

**Table 1:** Clinical characteristics of all invasive pulmonary aspergillosis cases.

Variable	Relapse (N = 14)	Non-relapse (N = 73)	*P*
**Age, median (IQR)**	55 (48–62)	53 (45–66)	0.721
**Male**	11 (78.6)	44 (60.3)	0.218
**Proven diagnosis (vs. Probable diagnosis)**	3 (21.4)	22 (30.1)	0.749
**Host factor**			
Main underlying diseases			
Haematologic disease^a^	11 (78.6)	49 (67.1)	0.535
Underwent stem cell transplantation	4 (28.6)	8 (11.0)	0.097
Solid organ transplantation	4 (28.6)	21 (28.8)	1.000
Others^b^	0 (0.0)	5 (6.8)	0.588
Additional underlying diseases			
Diabetes mellitus	9 (64.3)	25 (34.2)	0.035
Chronic lung disease	3 (21.4)	13 (17.8)	0.716
Charlson’s comorbidity-weighted index score, mean (± SD)	6.4 (± 1.4)	4.8 (± 2.3)	0.017
**Immunosuppressive events during initial treatment**			
Neutropenia	9 (64.3)	38 (52.1)	0.400
Graft-versus-host disease	1 (7.1)	6 (8.2)	1.000
Rejection	1 (7.1)	5 (6.8)	1.000
Any of above	11 (78.6)	44 (60.3)	0.233
***Aspergillus*** **antigen**			
Initial positivity^c^	10 (71.4)	55 (78.6)	0.727
*Aspergillus* antigen-positive week, median (IQR)^d^	2.4 (0.0–3.9)	1.5 (0.0–2.0)	0.462
**Duration of antifungal treatment**			
Voriconazole (weeks), median (IQR)	15.8 (8.2–21.5)	19.5 (12.0–22.9)	0.251
Short voriconazole treatment duration	4 (28.6)	7 (9.6)	0.063
All anti-mould agents (weeks), median (IQR)	17.3 (10.2–22.9)	21.9 (13.9–26.6)	0.210
Voriconazole/all anti-mould agents (%), median (IQR)	89.5 (86.1–100)	89.9 (84.2–100)	0.516
**Radiological findings**			
Initial			
Characteristics			
GGO with halo	11 (78.6)	58 (80.6)	1.000
Nodule/mass	11 (78.6)	57 (79.2)	1.000
GGO with consolidation	9 (64.3)	37 (51.4)	0.376
Cavitation	6 (42.9)	33 (45.8)	0.838
Centrilobular nodule	5 (35.7)	19 (26.4)	0.522
Number of involved lobes, median (IQR)	3 (2–5)	3 (2–5)	0.975
Sum of the three largest nodular sizes (cm), median (IQR)^e^	5.4 (3.6–6.6)	5.7 (2.7–7.4)	0.849
Radiological treatment response^f^			
Decreased number of involved lobes, median (IQR)	0 (0–1)	1 (0–2)	0.102
Decreased nodular size (cm), median (IQR)	1.8 (− 1.4 to 3.4)	2.8 (0.9–4.1)	0.246
Radiological response	4 (28.6)	42 (61.8)	0.023
Complete response	3 (21.4)	10 (14.7)	0.687

*IPA* invasive pulmonary aspergillosis, *IQR* interquartile range, *SD* standard deviation, *GGO* ground glass opacity.

^a^Including 35 cases of acute myelogenous leukemia, ten cases of acute lymphoid leukemia, six cases of myelodysplastic syndrome, three cases of lymphoma, and three cases of aplastic anaemia.

^b^Including two cases of lupus nephritis, one of dermatomyositis, one of hypereosinophilic syndrome, and one of alcoholic liver cirrhosis.

^c^After excluding three cases without *Aspergillus* antigen results.

^d^After excluding 15 cases without *Aspergillus* antigen follow-up results.

^e^After excluding 15 cases in which nodule size cannot be measured.

^f^After excluding five cases with no computed tomography scan results at the end of the treatment.

**Figure 2:**
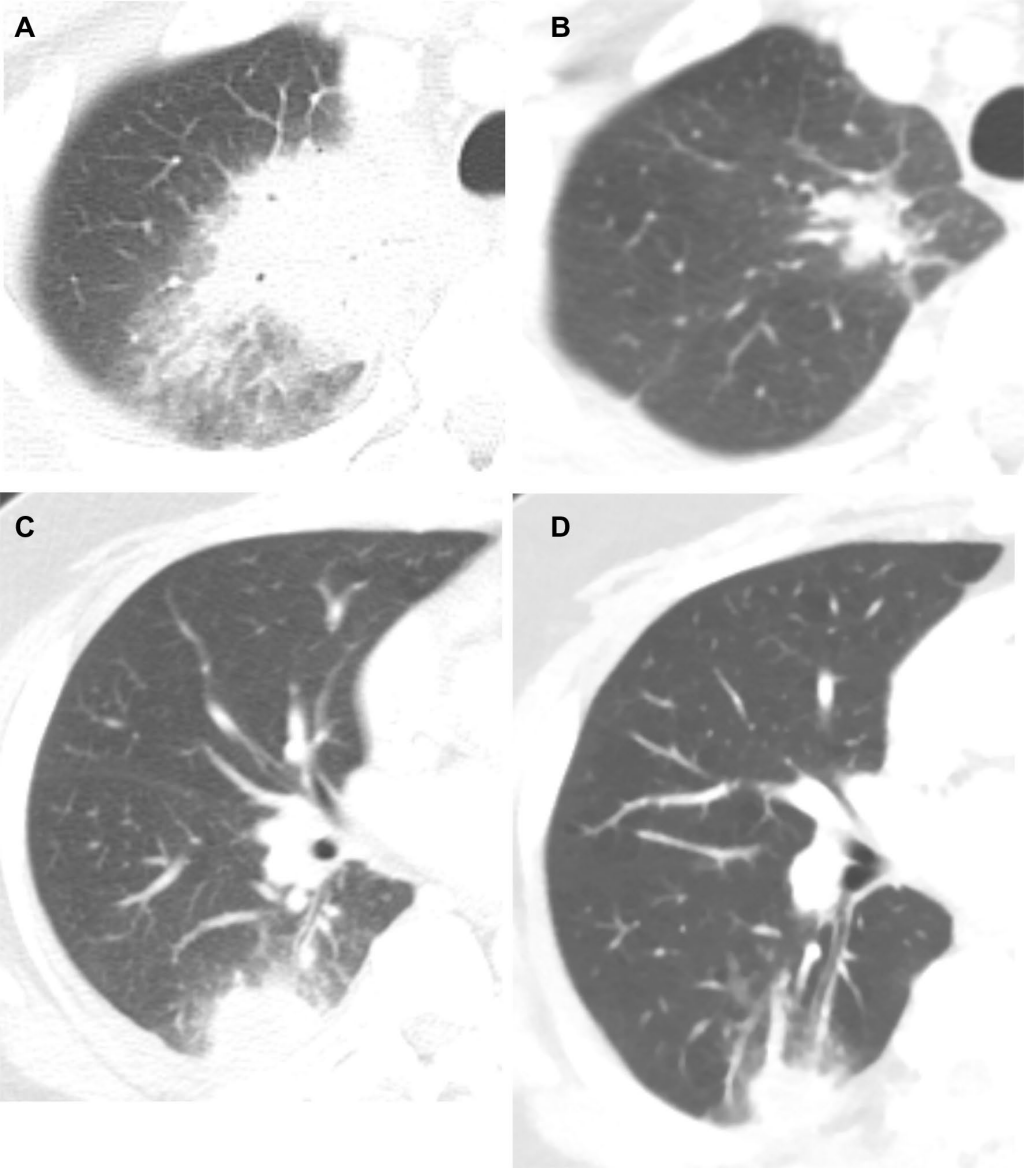
(**A**)–(**D**) Representative computed tomography findings from two patients. The first patient had a consolidation in the right upper lobe (**A**). A radiological response was achieved after initial treatment (**B**). The second patient had a nodule in the right lower lobe (**C**). A radiological response was not achieved (**D**). Invasive pulmonary aspergillosis relapsed in the second patient.

With respect to the radiological findings, the most common pattern of lung lesions was ground-glass opacity with a halo (78.6% vs. 80.6%; *P* = 1.000), followed by a nodule or mass (78.6% vs. 79.2%; *P* = 1.000) regardless of the relapse. The initial number of involved lobes was not significantly different between the two groups (median [IQR], 3 [2–5] vs. 3 [2–5]; *P* = 0.975). Radiological response (28.6% vs. 61.8%; *P* = 0.023) was significantly higher in the non-relapse group than in the relapse group, but there was no difference in the rate of complete response (CR) (21.4% vs. 14.7%; *P* = 0.687). Figure 2 shows representative CT findings from patients with (Fig. 2A,B) or without (Fig. 2C,D) a radiological response.

### Independent risk factors for IPA relapse

**Figure 3:**
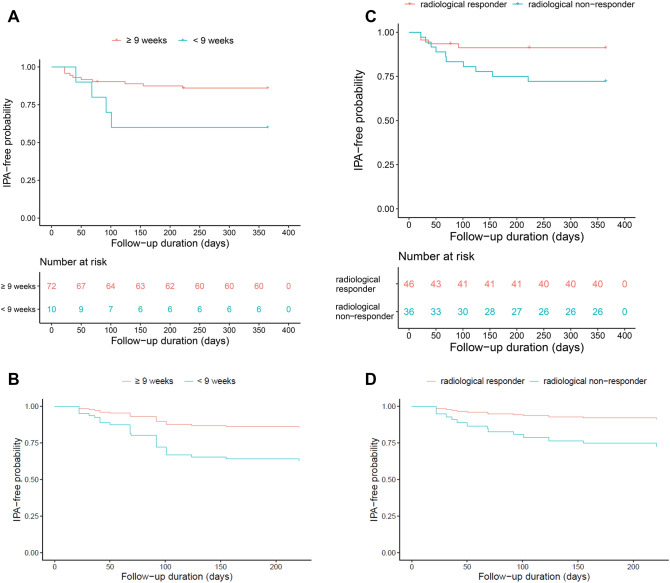
(**A**)–(**D**) Kaplan–Meier survival curves showing probability of being IPA-free based on duration of voriconazole therapy (**A**), and adjusted survival curves (**B**) after adjusting for data input into multivariable regression. Kaplan–Meier survival curves based on achievement of a radiological response after the end of the IPA treatment (**C**), and adjusted survival curves (**D**). *IPA* invasive pulmonary aspergillosis.

Multivariable Cox regression analysis identified that short voriconazole treatment duration (adjusted hazard ratio [aHR], 3.7; 95% confidence interval [CI], 1.1–12.3; *P* = 0.033; Table 2, Fig. 3A,B) and radiological non-response (aHR, 4.6; 95% CI, 1.2–17.5; *P* = 0.026; Fig. 3C,D) were associated with the relapse of IPA. Detailed results including aHR for the covariates are described in Table 2.

**Table 2:** Risk factors for relapse of invasive pulmonary aspergillosis.^a^

Variable	aHR (95% CI)	*P*
Age	0.9 (0.9–1.0)	0.096
Male	3.3 (0.8–13.0)	0.088
Charlson comorbidity-weighted index score	1.8 (1.2–2.6)	0.003
Number of initial involved lobes	1.1 (0.7–1.5)	0.777
Any immunosuppressive events during treatment	2.6 (0.7–10.3)	0.164
Short voriconazole treatment duration	3.7 (1.1–12.3)	0.033
Radiological non-response	4.6 (1.2–17.5)	0.026

*aHR* adjusted hazards ratio, *CI* confidence interval.

^a^After excluding five cases with no computed tomography scan results at the end of treatment.

### Sensitivity analyses

In the first sensitivity analysis, lack of radiological CR was not associated with relapse of IPA (aHR, 1.1; 95% CI: 0.2–5.8; *P* = 0.881). However, the aHR (3.9; 95% CI: 1.1–13.5; *P* = 0.031; Supplementary Table 1) for the short voriconazole treatment duration was significant. The second sensitivity analysis (n = 87), which excluded radiological response in the multivariable analysis, also identified that short voriconazole treatment was an independent risk factor (aHR, 3.9; 95% CI: 1.2–13.3; *P* = 0.028; Supplementary Table 2). The third sensitivity analysis (n = 72), which included duration of GM positivity instead of a radiological response in the regression, again revealed that the short treatment duration was a significant risk factor (aHR, 4.8; 95% CI: 1.1–21.3; *P* = 0.041; Supplementary Table 3).

## Discussion

In this study, we revealed that voriconazole treatment duration less than 9 weeks and radiological non-response at the time of treatment termination were associated with relapse of IPA. Our results imply that the voriconazole therapy should be continued for at least 9 weeks regardless of the underlying comorbidities in immunocompromised patients. Longer treatment may be indicated based on the patients’ symptoms, underlying medical conditions, and radiological improvement.

Defining appropriate treatment duration of IPA is difficult since it should be decided according to the immune status of the patients and treatment response^6^. Some guidelines suggest 6–12 weeks as the minimum duration of treatment although supporting evidence is weak^3^. A clinician should be prudent not to interpret these guidelines that the minimum of 6-week of treatment is sufficient since treatment duration less than 9-week could be risky according to the results of the present study.

Four patients with short voriconazole duration experienced relapse, and the intervals from the end of voriconazole treatment to the diagnosis of relapse in these patients were 41, 68, 92, 101 days, respectively (Fig. 3A). Although it was not possible to molecularly distinguish between relapse and reinfection in the present study^8,9^, such a short interval between the end of the treatment and re-diagnosis supports that it was a relapse rather than reinfection.

The reliable radiological predictors of treatment of IPA remain unknown. According to Vehrechild et al., an increase of IPA burden in serial chest CT in early course of treatment was a sensitive marker of poor prognosis^10^. In this study, we investigated the radiological responses using definitions from previous study^1^, and revealed that the failure to achieve more than 50% reduction of IPA burden was significantly associated with relapse. If the radiological response is not achieved at the time when a clinician had planned to terminate the treatment, an additional course of voriconazole should be considered.

Sensitivity analyses consistently demonstrated that the short voriconazole treatment duration less than 9 weeks was associated with relapse of IPA. Severe underlying illness, a well-known risk factor associated with poor prognosis^11,12^, was also associated with relapse. Interestingly, radiological CR was not an independent risk factor. However, it is understandable given the fact that only 13 (15.9%) patients achieved radiological CR in our cohort. In this regard, we assume that the radiological response, which includes a broader range of radiological improvement than radiological CR, would be a more appropriate marker to assess the treatment response.

For accurate assessment of aHRs for the variables of interest, we included severity of underlying diseases, immunosuppressive events during IPA treatment, and initial burden of IPA as covariates in the multivariable analysis. These covariates are known as poor prognostic factors of IPA. A retrospective study of 385 patients with invasive aspergillosis and various underlying conditions showed that receipt of allogeneic HCT or solid-organ transplantation, renal impairment, neutropenia, and diffuse pulmonary lesions were predictors of increased overall mortality^11^. Another study of 405 cases of invasive aspergillosis after HCT identified low pulmonary function, neutropenia, and elevated bilirubin or creatinine levels as independent risk factors for mortality^12^. Finally, a retrospective study of 27 invasive aspergillosis cases after bone marrow transplantation identified acute or extensive graft-versus-host disease (GVHD) as a risk factor for mortality^13^.

This study has several limitations. First, it was a retrospective study conducted at a single tertiary-care hospital with a limited number of patients with IPA. Therefore, the results should be validated using a larger prospective cohort. Second, eligible patients had often received long-term voriconazole treatment. Although this may be partly due to unresolved underlying disease, it may have lowered the relapse rate of IPA in our cohort. Third, since the prognosis of IPA depends on the severity of immunosuppression^3,5^, levels of immunosuppression after initial treatment may be associated with relapse. However, we did not include immunosuppressive events after treatment in the regression analysis as i) the immunosuppressive events during and after treatment are highly correlated with each other, and ii) the variable is not available for a clinician at the time of the treatment termination. Finally, we could not derive an optimal treatment duration for IPA from our study results. Further prospective studies with a larger cohort are warranted to define the optimal duration of therapy or to develop and validate a prediction model to personalize the treatment duration.

In conclusion, short duration of voriconazole therapy and post-treatment radiological non-response were independent risk factors for relapse of IPA. Therefore, voriconazole treatment should be continued for at least 9 weeks in immunocompromised patients and the radiological response should be verified closely at the time of treatment termination.

## Methods

### Study design

All adults (age $\ge$ 18 years) with proven or probable IPA^14^ who had finished voriconazole treatment between January 2005 and July 2019 at Seoul National University Hospital were retrospectively reviewed. Patients who died or were lost to follow-up during initial treatment or within 1 year after the end of the treatment were excluded. Case and control patients comprised those that suffered IPA relapse and those that did not, respectively. The study was approved by the Seoul National University Hospital Institutional Review Board (No. 1909-011-1061). The requirement for written informed consent was waived due to the retrospective nature of the study. All methods were carried out in accordance with the approved guidelines.

### Clinical characteristics

The following clinical data were collected from electronic medical records: age, sex and underlying disease (e.g., haematologic diseases [acute myelogenous leukaemia, myelodysplastic syndrome and history of HCT], solid-organ transplantation, or connective tissue disease, and history of diabetes mellitus or chronic lung disease). The severity of comorbidities was measured using the Charlson’s comorbidity-weighted index score^15,16^. It was also reviewed if the patient had experienced severe neutropenia, GVHD, or rejection during treatment for IPA. The duration of voriconazole or other anti-mold treatments was also noted.

Serum GM was measured using an enzyme-linked immunosorbent assay (Platelia *Aspergillus* kit) according to the manufacturer’s instructions (Bio-Rad Laboratories, Hercules, CA, USA). The threshold optical density index of *Aspergillus* galactomannan was set at 0.5^17^. *Aspergillus* antigen-positive week, the period until GM titres became less than 0.5, was reviewed.

Chest CT scans were reviewed at the start and end of antifungal treatment, and the characteristics (i.e., ground-glass opacity, consolidation or mass with or without cavitation) and burden of IPA lesions were noted. The sums of the longest diameters of up to three lung nodules, the number of nodules and the number of involved lobes were recorded as surrogates for IPA burden. Also, changes in IPA burden before and after the treatment were reviewed.

### Definitions

A definition of proven or probable IPA was made in accordance with the revised criteria of the European Organization for Research and Treatment of Cancer/Mycoses Study Group^14^. Relapse was defined as a re-diagnosis of proven or probable IPA at the same site with or without dissemination within 1 year after the end of initial voriconazole treatment, according to Cornely et al*.*^18^. If initial lesions were not completely resolved at treatment termination, aggravation with additional evidence of proven or probable IPA (positive GM or culture with appropriate clinical context) was regarded as a relapse. Time from the end of treatment to diagnosis of relapse was calculated.

Neutropenia was defined as an absolute neutrophil count of < 500/μL for more than 1 week^19,20^. Definitions of GVHD^21,22^ and rejection^23–25^ in patients who underwent HCT or solid-organ transplantation were the same as in previous reports. Radiological response was defined as more than 50% reduction in the IPA burden without aggravation of original lesions or occurrence of new lesions^1^. Radiological CR was defined as disappearance of all lung lesions. Duration of voriconazole treatment was dichotomized arbitrarily into two groups, with 9 weeks as the threshold (i.e., median value of commonly recommended duration by the Infectious Disease Society of America^3^).

### Statistical analysis

The clinical and microbiological characteristics of patients with and without IPA relapse were compared. According to results of the Shapiro–Wilk test, the Student’s *t*-test or Mann–Whitney *U-*test was used to compare continuous variables. The Chi-squared test or Fisher’s exact test was used to compare categorical variables.

Multivariable Cox regression analysis was conducted to identify independent risk factors for relapse after treatment^26^. Duration from the end of initial treatment to diagnosis of relapse was used as a time variable. Inclusion of variables in multivariable analyses was based on a priori clinical knowledge of the risk factors for poor prognosis in patients with IPA^27,28^. Specifically, impaired renal or liver function^11,12^; immunosuppressive events such as neutropenia, GVHD or rejection^11,13,29,30^; and the number of lung lobes involved initially^11^ were analyzed as covariates, in addition to radiological treatment responses^10^ and duration of voriconazole treatment. Multicollinearity was checked using variance inflation factors, with 5 set as the cut-off. Unadjusted Kaplan–Meier survival curves were drawn after identification of the independent risk factors. In addition, a marginal approach with reweighted data was used to construct adjusted survival curves^31^.

Sensitivity analyses were performed in three ways. First, multivariable analysis was performed using CR rather than the whole radiological response as a treatment response variable. Second, to eliminate confounding effects introduced by patients lacking follow-up CT scans, analysis was conducted after excluding the radiological response. Lastly, *Aspergillus* antigen-positive week was added instead of a radiological response in the regression analysis as a surrogate for the therapeutic effect.

All analyses were performed using PASW for Windows (version 25.0; SPSS Inc., Chicago, IL, USA). Survival curves were plotted using R software version 3.5.1 (https://www.R-project.org; survminer package). A *P* value < 0.05 was considered statistically significant.

## Supplementary information

Supplementary Information.

## Data Availability

The data supporting this publication can be accessed by contacting the corresponding authors on reasonable request.
