# Supplementary material for: Short course of voriconazole therapy as a risk factor for relapse of invasive pulmonary aspergillosis
Source: Sci Rep. 2020 Sep 30;10:16078. doi: 10.1038/s41598-020-73098-w (PMC7527978; doi:10.1038/s41598-020-73098-w)
Supplement: Supplementary file 1 — Supplementary Information. [file 41598_2020_73098_MOESM1_ESM.docx]

**Short Course of Voriconazole Therapy as a Risk Factor for Relapse of Invasive Pulmonary Aspergillosis**

Dong Hoon Shin^1,*^, Seung-Jin Yoo^2,*^, Kang Il Jun^1^, Hyungjin Kim^2,†^, Chang Kyung Kang^1,†^, Kyoung-Ho Song^1,3^, Pyoeng Gyun Choe^1^, Wan Beom Park^1^, Ji-Hwan Bang^1,4^, Eu Suk Kim^1,3^, Sang Won Park^1,4^, Hong Bin Kim^1,3^, Nam-Joong Kim^1^, and Myoung-don Oh^1^

^1^ Department of Internal Medicine, Seoul National University College of Medicine, Seoul, Republic of Korea. ^2^ Department of Radiology, Seoul National University Hospital, Seoul, Republic of Korea. ^3^ Department of Internal Medicine, Seoul National University Bundang Hospital, Seongnam, Republic of Korea. ^4^ Department of Internal Medicine, Seoul Metropolitan Boramae Hospital, Seoul, Republic of Korea.

* DHS and S-JY contributed equally to this work.

† Corresponding authors: HK, [khj.snuh@gmail.com](mailto:khj.snuh@gmail.com) and CKK, [zeptemiger@hanmail.net](mailto:zeptemiger@hanmail.net)

**Supplementary Table 1.** Risk factors for relapse of invasive pulmonary aspergillosis, including a complete response.^a^

| Variable | aHR (95% CI) | *P* |
| --- | --- | --- |
| **Age** | 1.0 (0.9–1.0) | 0.262 |
| **Male** | 2.8 (0.7–11.0) | 0.132 |
| **Charlson comorbidity-weighted index score** | 1.7 (1.1–2.4) | 0.009 |
| **Number of initial involved lobes** | 1.2 (0.8–1.7) | 0.459 |
| **Any immunosuppressive events during treatment** | 3.5 (0.9–13.7) | 0.076 |
| **Short voriconazole treatment duration** | 3.9 (1.1–13.5) | 0.031 |
| **Lack of complete response** | 1.1 (0.2–5.8) | 0.881 |

aHR: adjusted hazards ratio; CI: confidence interval.

^a^ After excluding five cases with no computed tomography scan results at the end of treatment.

**Supplementary Table 2.** Risk factors for relapse of invasive pulmonary aspergillosis, excluding a radiological response.

| Variable | aHR (95% CI) | *P* |
| --- | --- | --- |
| **Age** | 1.0 (0.9–1.0) | 0.192 |
| **Male** | 2.6 (0.7–9.8) | 0.154 |
| **Charlson comorbidity-weighted index score** | 1.7 (1.2–2.4) | 0.006 |
| **Number of initial involved lobes** | 1.2 (0.8–1.7) | 0.388 |
| **Any immunosuppressive events during treatment** | 3.4 (0.9–13.4) | 0.075 |
| **Short voriconazole treatment duration** | 3.9 (1.2–13.3) | 0.028 |

aHR: adjusted hazards ratio; CI: confidence interval.

**Supplementary Table 3.** Risk factors for relapse of invasive pulmonary aspergillosis, including the galactomanann assay instead of a radiological response.^a^

| Variable | aHR (95% CI) | *P* |
| --- | --- | --- |
| **Age** | 1.0 (0.9–1.0) | 0.226 |
| **Male** | 2.3 (0.5–9.5) | 0.261 |
| **Charlson comorbidity-weighted index score** | 1.7 (1.1–2.5) | 0.009 |
| **Number of initial involved lobes** | 1.0 (0.6–1.5) | 0.837 |
| **Any immunosuppressive events during treatment** | 2.3 (0.5–10.2) | 0.266 |
| **Short voriconazole treatment duration** | 4.8 (1.1–21.3) | 0.041 |
| ***Aspergillus* antigen-positive week** | 1.0 (0.9–1.3) | 0.638 |

aHR: adjusted hazards ratio; CI: confidence interval.

^a^ After excluding 15 cases without *Aspergillus* antigen follow-up results.
